# Intercellular diffusion of cyclic nucleotides followed by gap junction closure restarts meiosis in mouse preovulatory follicles

**DOI:** 10.1073/pnas.2524136122

**Published:** 2025-12-02

**Authors:** Iris F. Nakashima, Haining Zhong, Viacheslav O. Nikolaev, Corie M. Owen, Siu-Pok Yee, Laurinda A. Jaffe, Jeremy R. Egbert

**Affiliations:** ^a^Department of Cell Biology, University of Connecticut Health Center, Farmington, CT 06030; ^b^Vollum Institute, Oregon Health and Science University, Portland, OR 97239; ^c^Institute of Experimental Cardiovascular Research, University Medical Center Hamburg-Eppendorf, Hamburg D-20246, Germany; ^d^Center for Mouse Genome Modification, University of Connecticut Health Center, Farmington, CT 06030

**Keywords:** ovarian follicle, luteinizing hormone, cyclic nucleotides, gap junctions, oocyte meiosis

## Abstract

Signaling by luteinizing hormone (LH) in the outer granulosa cells of mammalian ovarian follicles causes meiosis to resume in the oocyte, located ~10 cell layers away, preparing the oocyte for ovulation and fertilization. This long-distance communication is accomplished by cAMP and cGMP diffusion through gap junctions, but knowledge of cAMP dynamics in the oocyte is based on static measurements, and information about cAMP changes in the granulosa cells has not been integrated with information about cAMP changes in the oocyte. By simultaneous multihour imaging of both compartments of live ovarian follicles, using mice expressing an improved cAMP sensor, we elucidate how the meiosis-activating signal is transmitted. In response to LH, cAMP generated in the granulosa cells diffuses within ~10 min to the oocyte. cAMP in the granulosa cells then remains high for at least 5 h, but over a 3-h period, cAMP in the oocyte decreases to a new plateau level below the original baseline. We show that the cAMP decrease in the oocyte depends not only on the established mechanism of LH lowering cGMP in the oocyte, which relieves inhibition of the PDE3A phosphodiesterase in the oocyte, but also on the subsequent LH-induced closure of gap junctions between the granulosa cells. This closure prevents cAMP from diffusing into the oocyte from the granulosa cells, a concept that has been proposed but not previously tested. We conclude that LH coordinates changes in both cGMP and gap junctions to lower cAMP in the oocyte, reinitiating meiotic progression.

## Significance

Continuous time-lapse imaging of cyclic AMP and cyclic GMP in mouse ovarian follicles elucidates how luteinizing hormone (LH) regulation of cyclic GMP and gap junctions in the somatic cells surrounding the oocyte transmits a signal that lowers cyclic AMP in the oocyte and reinitiates meiotic progression in preparation for ovulation and fertilization.

Each oocyte within a mammalian ovary is at the center of a communicating network of granulosa cells; this complex is known as an ovarian follicle (1). The oocytes are arrested in prophase of meiosis, part way through the process that will eventually reduce the number of each chromosome to one, such that the female and male genomes can combine to produce a new individual at fertilization. With each reproductive cycle, one or more follicles grow to the preovulatory stage, with the granulosa cells proliferating to form ~10 cellular layers and acquiring receptors for luteinizing hormone (LH) in the outer layer (see ref. 2). LH is released from the pituitary gland, over a period of several hours (3), and acts on these receptors to generate the signals that restart meiosis, cause ovulation, and transform the follicle into the corpus luteum that supports pregnancy (4). The long-distance communication of the signal that restarts meiosis is accomplished by cAMP and cGMP diffusion through gap junctions (see 5), but knowledge of cAMP dynamics in the oocyte is based on static measurements (6), and spatial and temporal changes in cAMP in the granulosa cells (7) have not been integrated with cAMP changes in the oocyte.

Oocytes within preovulatory follicles are maintained in meiotic prophase by a high level of cAMP (8, 9), which is generated under the control of a constitutively active G_s_-coupled receptor, GPR3, in the oocyte (9). cAMP is also generated in the granulosa cells under the control of the G_s_-coupled receptor for follicle-stimulating hormone (FSH) (4), but cAMP from the granulosa cells is insufficient to maintain meiotic arrest in the absence of GPR3 in the oocyte (10). The high level of oocyte cAMP is maintained by cGMP that diffuses into the oocyte from the surrounding granulosa cells through gap junctions and competitively inhibits the PDE3A phosphodiesterase in the oocyte (6). The cGMP is generated by the guanylyl cyclase NPR2 in the granulosa cells (11, 12).

LH signaling lowers cGMP in the granulosa cells, primarily by dephosphorylating and inactivating NPR2, thus reducing the synthesis of cGMP by the granulosa cells (13–17). Even before LH stimulation, the granulosa cells have cGMP phosphodiesterase activity, due in part to PDE5A, and LH stimulation slightly increases PDE5A activity as a consequence of phosphorylating the PDE5A protein (18). The net effect of decreased cGMP production by NPR2, in the presence of cGMP hydrolyzing activity, is to lower cGMP in the granulosa cells. When cGMP falls in the granulosa cells, cGMP diffuses out of the oocyte, via gap junctions, into the large volume of the granulosa cell cytoplasm, decreasing cGMP in the oocyte. When cGMP falls in the oocyte, competitive inhibition of the PDE3A phosphodiesterase in the oocyte is relieved, increasing hydrolysis of cAMP, and thus decreasing the concentration of cAMP in the oocyte (6).

However, it has not been directly tested whether preventing the LH-induced cGMP decrease in the granulosa cells prevents the cAMP decrease in the oocyte. It is also unknown if the decrease in cGMP is the only factor that causes the cAMP decrease. LH signaling transiently decreases the permeability of the gap junctions between the granulosa cells (19), but it is unknown whether this permeability decrease plays a part in lowering oocyte cAMP. Paradoxically, the decrease in oocyte cAMP is initiated by an LH-induced increase in cAMP in the surrounding granulosa cells, which persists for at least several hours (7, 20–22). An improved fluorescent sensor for cAMP, cAMPFIRE-M (23), used together with the cGi500 sensor for cGMP (13, 24, 25), now allows us to integrate these complex components of LH signaling in the ovary, explaining how LH causes meiosis to resume in the oocyte.

## Results and Discussion

### Before LH Stimulation, cAMP Levels in Oocytes and Granulosa Cells of Preovulatory Follicles Are Similar.

**Fig. 1.**
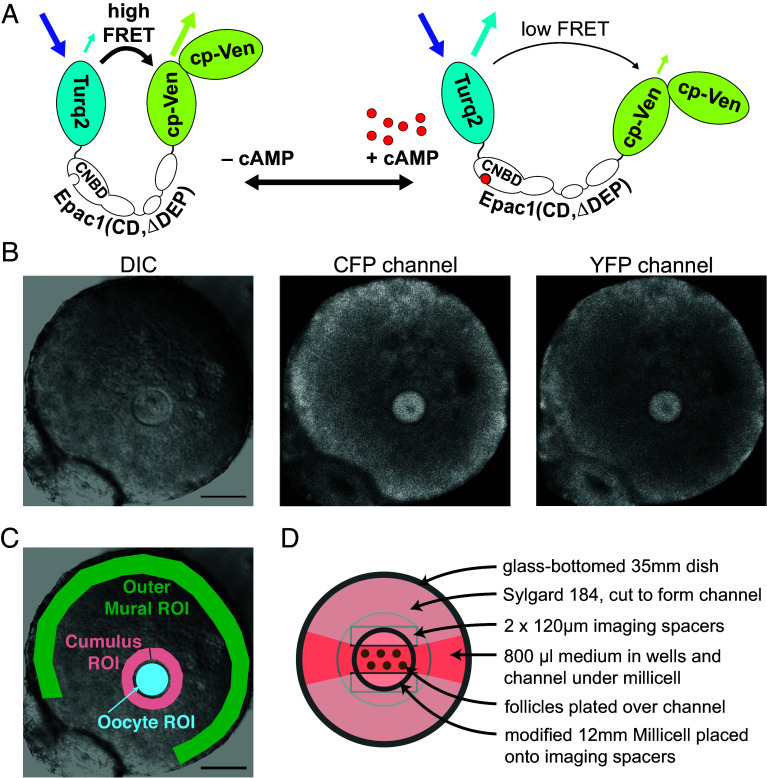
Measurement of cAMP dynamics in follicles and follicle-enclosed oocytes expressing cAMPFIRE-M. (*A*) Diagram of cAMPFIRE-M, composed of modified fluorescent proteins that emit cyan or yellow light (hereafter called “CFP” and “YFP”, respectively) that are coupled by a modified version of the cAMP-binding protein EPAC1; cAMP binding decreases FRET. Turq2: mTurquoise2 with C-terminal truncation of 11 amino acids (mTurquoise2Δ); cp-Ven: circularly permuted variant of Venus (^cp173^Venus); CNBD: cyclic nucleotide binding domain of EPAC1. CD: catalytically dead mutant of EPAC1. ΔDEP: deletion of the EPAC1 Disheveled, Egl-10, and Pleckstrin domain to prevent membrane localization. (*B*) Follicle from a cAMPFIRE-M expressing mouse; additional cAMPFIRE-M protein was expressed in the oocyte by microinjection of cAMPFIRE-M mRNA 18 h previously. The DIC image shows the centrally located oocyte surrounded by granulosa cells, with a thin layer of theca cells at the periphery. The prophase-arrested nucleus and nucleolus (germinal vesicle) is visible near the center of the oocyte, while the oil droplet from the microinjection is seen slightly out of focus adjacent to the nucleus at ~11 o’clock. In the fluorescence images, the oocyte nucleus is visible as a dark circle lacking cAMPFIRE-M fluorescence. Dark areas in the granulosa region are antral space, appearing as patches due to some flattening of the follicle on the Millicell. (Scale bar, 100 µm.) (*C*) Representative regions of interest (ROIs) for the outer mural granulosa cells (~40 µm wide band, green), cumulus cells (~25 µm wide band, pink), and the oocyte (blue). (*D*) Diagram of imaging dish with follicles on a modified Millicell adhered to a glass-bottomed culture dish using rectangular imaging spacers, forming a channel under the center of the Millicell. Sylgard dams around the Millicell form wells of medium on either end of the channel. Multiple follicles can be placed on the Millicell, allowing imaging of the entire set during a single time series.

To image the spatiotemporal dynamics of LH-induced changes in cAMP throughout all regions of the preovulatory follicle over the several hours leading to meiotic resumption in the oocyte, 3 advances were essential. 1) We used the cAMPFIRE-M sensor (23) (Fig. 1*A*), which showed an ~5X larger change in CFP to YFP FRET in response to LH compared with the original Epac-camps sensors (26) used for previous measurements of cAMP dynamics in ovarian follicles (6, 7) (*SI Appendix*, Table S1). 2) We expressed the sensor in both the oocyte and granulosa cells within the intact follicle (Fig. 1 *B* and *C*). 3) We maintained the follicles in physiological conditions with a stable focal plane during multihour imaging and medium exchange (Fig. 1*D*).

**Fig. 2.**
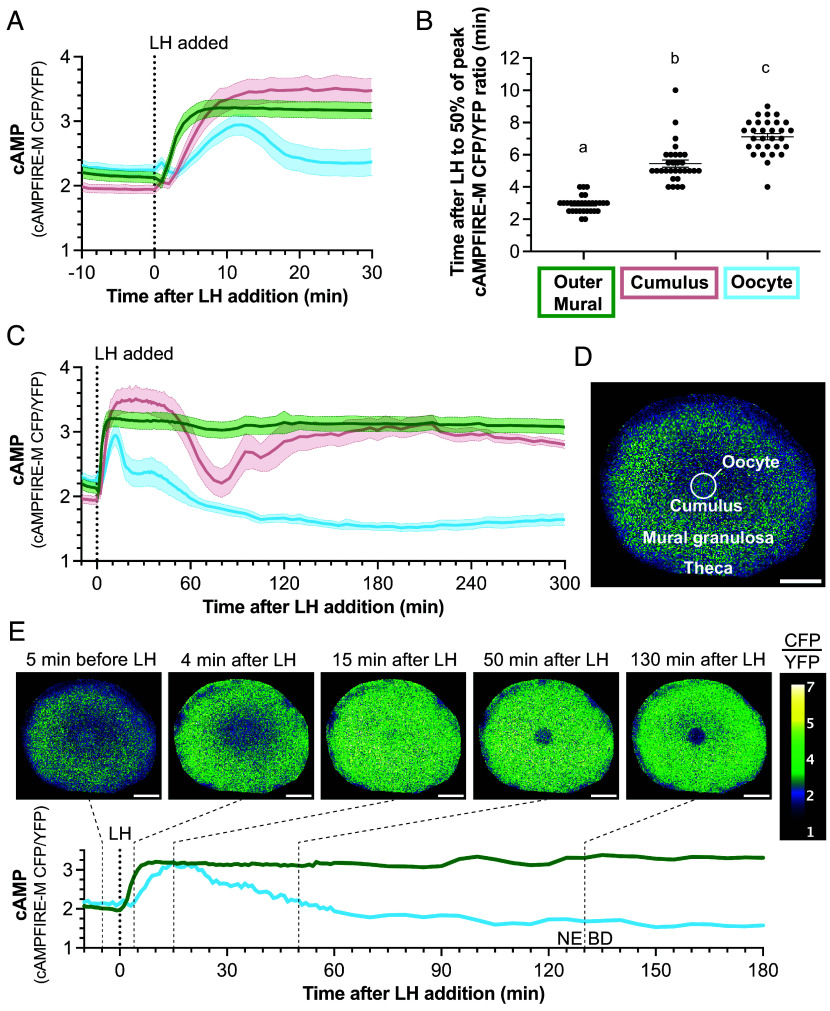
Kinetics of LH-induced changes in cAMP in the mural granulosa cells, cumulus cells, and oocyte of preovulatory follicles. (*A* and *B*) Progression of the LH-induced cAMP elevation from outer mural granulosa (green) to cumulus (pink) to oocyte (blue). (*A*) shows the CFP/YFP emission ratio for cAMPFIRE-M in each region as a function of time after LH addition (mean ± 95% confidence limit; n = 30 follicles). The CFP/YFP emission ratio is approximately proportional to the log of the cAMP concentration, within the linear range of the concentration curve (*SI Appendix*, Fig. S1*B*). (*B*) shows the time to reach 50% of the peak ratio: 2.9 ± 0.1 min in outer mural, 5.5 ± 0.2 min in cumulus, 7.1 ± 0.2 min in oocyte (mean ± SEM). Different letters indicate significant (*P* < 0.0001) differences between groups (one-way repeated measures ANOVA with the Holm–Sidak correction for multiple comparisons). (*C*) LH-induced changes in cAMP over a 5-h period in the three measured regions. Data for the first 30 min are replotted from *A*; n = 30 follicles. (*D* and *E*) Images from Movie S1. (*D*) shows the follicle before LH addition, with CFP/YFP emission ratios from cAMPFIRE-M displayed in 5.6 µm^2^ pixels within the optical section. E shows the follicle at selected time points after LH addition, corresponding to the records from the granulosa and oocyte below. Nuclear envelope breakdown (NEBD) had just occurred in the last image at 130 min after LH. (Scale bar, 100 µm.)

Before LH addition, CFP/YFP emission ratios measured in the granulosa cell and oocyte regions of the follicle were similar, indicating that the average basal cAMP concentrations within these regions are similar (Fig. 2*A*). Using a calibration method like that described previously (6), we estimated that this basal cAMP concentration is approximately ~700 nM (*SI Appendix*, Fig. S1). This value is similar to that determined using Epac2-camps300 in follicle-enclosed oocytes (6). Based on in vitro–determined activation constants for regulatory subunits of the cAMP-activated protein kinase (PKA) of ~30-600 nM (27), a concentration of ~700 nM cAMP in the oocyte is consistent with evidence that PKA is active in the oocyte and maintains meiotic arrest (28). In contrast, PKA activity in the granulosa cell compartment prior to LH exposure is low, based on low levels of PKA-dependent phosphorylation of CREB and other substrates in whole follicles (17). Thus, the relatively high cAMP concentration that our measurements indicate for the granulosa cells is surprising.

This apparent discrepancy could be due to the PKA regulatory subunit protein concentrations in the granulosa cells being higher than the regulatory subunit concentrations used in vitro to determine PKA activation constants (29, 30). Additionally, cAMP concentrations in the subcellular compartments where the PKA protein is located could be lower than the average value for the cytosol as a whole (31–33). Measurements of PKA activity using AKAR sensors (31, 34) expressed in living follicles could provide insight into how similar average cAMP concentrations can differentially affect PKA activity in granulosa cells and oocyte.

### In Response to LH, cAMP Increases in All Compartments of the Follicle, Then Stays High in the Mural Layer While Decreasing to Below Baseline in the Oocyte.

In the initial ~10 min after applying LH to the follicle, cAMP increased throughout the tissue: first in the outer mural granulosa cells where the LH receptors are located, then in the cumulus granulosa cells directly around the oocyte, then in the oocyte (Fig. 2 *A* and *B*; regions as defined in Fig. 1*C*). The propagation of the cAMP increase from the mural cells to the cumulus was attenuated by the gap junction blocker carbenoxolone, with the change in cAMPFIRE-M CFP/YFP emission ratio after addition of LH reduced to ~30% of control (*SI Appendix*, Fig. S2; see also 7). The propagation to the oocyte was almost completely inhibited by carbenoxolone (*SI Appendix*, Fig. S2). The time for the increase in cAMP to spread from the mural granulosa cells to the oocyte was approximately the same as the time required for diffusion between these 2 compartments of a fluorescent tracer with a similar molecular weight to cAMP (19). We concluded that the LH-induced cAMP increase in the oocyte occurs at least mostly by diffusion through gap junctions.

Over the next 5 h, cAMP remained at a constant high level in the mural granulosa cells (Fig. 2*C*). However, in the oocyte, the cAMP increase was transient, falling to below baseline after about 1 h, and continuing to decline to a minimum at about 3 h after LH application (Fig. 2*C*). Based on the calibration curve shown in *SI Appendix*, Fig. S1, the minimum cAMP concentration in the oocyte was ~170 nM, compared to ~700 nM before LH application. The maximum cAMP concentration attained in the mural granulosa cells was ~3,400 nM (*SI Appendix*, Fig. S1).

In the cumulus region, the increase in cAMP was followed by a transient decrease between about 1 and 2 h, then a return to a high level like that in the mural cells (Fig. 2*C*). cAMP kinetics in the cumulus region were variable, and about half of our 30 records showed additional oscillations (*SI Appendix*, Fig. S3).

Movie S1 shows an example of the spatial dynamics of the cAMP changes in the follicle over the 3 h after LH addition, and images from the first 2 h of this video summarize the changes in the mural granulosa cells and oocyte (Fig. 2*D*). Before LH exposure, the average cAMP concentration was similar in both compartments. At 4 min after applying LH, cAMP was elevated in the mural granulosa but not in the oocyte. At 15 min, cAMP was elevated in both compartments. At 50 min, cAMP remained high in the mural granulosa but had decreased to baseline in the oocyte. At 130 min, cAMP was still high in the mural granulosa but had decreased to below baseline in the oocyte. Movie S1 also illustrates the multiple oscillations in cAMP that were sometimes seen in the cumulus region.

Outside of the granulosa compartment of a follicle, there is a basal lamina, and beyond that several heterogeneous layers of theca cells (Fig. 1*B*) that are not connected to the granulosa cells by gap junctions (19). A variable amount of theca remains with the follicle after isolation. The basal cAMP concentration in the theca region was lower than in the granulosa (Fig. 2*D*). LH caused cAMP to increase in some of the theca cells (Fig. 2*E* and Movie S1), some of which express LH receptors (2). Because dissection of follicles from the ovary pulls apart the theca layer, leaving an inconsistent fraction of the theca associated with the isolated follicles, LH responses in these cells were not analyzed further.

### Inhibiting the LH-Induced GMP Decrease Inhibited the cAMP Decrease in the Oocyte.

**Fig. 3.**
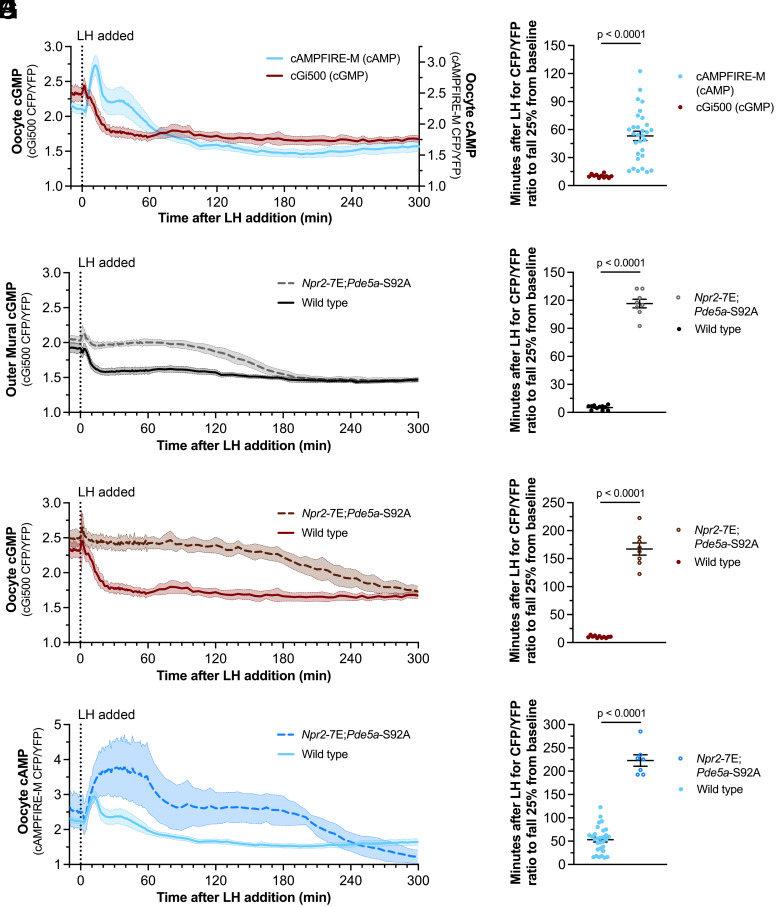
The LH-induced fall in oocyte cAMP is preceded by a fall in oocyte cGMP and is delayed by delaying the GMP decrease. (*A*) Temporal relationship between the LH-induced cGMP (brown) and cAMP (blue) decreases in the oocyte. n = 11 cGi500 oocytes. The cAMPFIRE-M data are replotted from Fig. 2*C*. (*B*) Comparison of the kinetics of the initiation of cGMP and cAMP decreases in the oocyte, measured from A. Y-axis values indicate the time after LH at which the CFP/YFP emission ratio has decreased by 25% from the baseline before LH to the minimum level after LH. (*C*–*F*) Preventing NPR2 dephosphorylation and PDE5A phosphorylation delays the LH-induced cGMP decreases in the outer mural granulosa cells (*C* and *D*) and oocytes (*E* and *F*) compared to wild type (n = 11 wild-type follicles; n = 8 *Npr2*-7E; *Pde5a*-S92A follicles). Wild-type data in E replotted from Fig. 3*A*. Note that the inhibition of the LH-induced cGMP decrease in the oocyte by the *Npr2*-7E; *Pde5a*-S92A mutation (*E*) is more complete than reported in ref. 18; this may be because in the previous study the concentration of LH was 30× higher. (*G* and *H*) The LH-induced decrease in oocyte cAMP is delayed in *Npr2*-7E; *Pde5a*-S92A mice (n = 30 wild-type follicles; n = 7 *Npr2*-7E; *Pde5a*-S92A follicles). Wild-type data replotted from Fig. 2*C*. Traces are plotted as mean ± 95% confidence limit; points are plotted as mean ± SEM. Unpaired *t* tests with Welch’s correction for unequal variances.

Previous studies have indicated that a primary cause of the LH-induced cAMP decrease in the oocyte is the LH-induced decrease in cGMP in the oocyte, which relieves the inhibition of PDE3A, resulting in cAMP hydrolysis (6). These studies showed that lowering cGMP in follicle-enclosed oocytes by injection of a cGMP-specific phosphodiesterase lowered oocyte cAMP (6). In support of this mechanism, the LH-induced cGMP decrease in the oocyte, as measured with the cGMP-specific FRET sensor cGi500, preceded the LH-induced cAMP decrease in the oocyte, as measured with cAMPFIRE-M (Fig. 3 *A* and *B*). However, it has not been directly tested whether inhibiting the LH-induced cGMP decrease inhibits the cAMP decrease in the oocyte.

To test this hypothesis, we used mice in which the LH-induced cGMP decrease in the mural granulosa cells was inhibited by mutations that prevent LH-induced dephosphorylation and inactivation of the NPR2 guanylyl cyclase (*Npr2*-7E) and LH-induced phosphorylation and activation of the cGMP phosphodiesterase PDE5A (*Pde5a*-S92A) (18). In follicles from these mice, the LH-induced fall in mural granulosa cell cGMP was delayed (Fig. 3 *C* and *D*), with the time to reach the minimum level extended from ~20 min to ~3 h (Fig. 3*C*).

The eventual decrease in mural cell cGMP corresponds temporally with a gradual LH-induced decrease in the follicle content of C-type natriuretic peptide (CNP, also known as NPPC), an agonist that is required for NPR2 guanylyl cyclase activity (14, 35, 36). In the presence of exogenously added CNP, cGMP in the mural cells remained relatively stable for the duration of the 5-h recording (*SI Appendix*, Fig. S4), supporting the conclusion that the delayed cGMP decrease 2 to 3 h after LH application to *Npr2*-7E;*Pde5a*-S92A follicles is due primarily to the LH-induced decrease in follicle CNP content.

As a consequence of the delay in the LH-induced decrease mural granulosa cGMP (Fig. 3*C*), the fall in oocyte cGMP to the same level as in wildtypes was delayed by several hours in the *Npr2-7E; Pde5a*-S92A mice (Fig. 3 *E* and *F*). The cAMP decrease in the oocyte was similarly delayed (Fig. 3 *G* and *H*), indicating that the LH-induced cGMP decrease is essential for the cAMP decrease. Consistent with the delayed decrease in oocyte cAMP, the reinitiation of meiosis, as marked by nuclear envelope breakdown (NEBD) and disappearance of the nucleolus, also known as the germinal vesicle breakdown, is delayed by ~5 h in follicles from *Npr2*-7E mice (15).

### Gap Junction Closure Is Essential for the Normal Kinetics of the LH-Induced Oocyte cAMP Decrease and Meiotic Resumption.

In addition to the high concentration of cGMP in the granulosa cells, gap junction permeability between the cells of the follicle is required to maintain meiotic arrest (19, 37, 38), and it has been proposed that an LH-induced decrease in gap junction permeability could facilitate the LH-induced decrease in cAMP in the oocyte (39–41). LH signaling causes a partial and transient closure of the gap junctions between the granulosa cells (19), but whether this change is sufficient to contribute to the cAMP decrease in the oocyte has not been tested.

The permeability of the gap junctions between the granulosa cells, which are comprised of connexin 43 (Cx43 or GJA1), begins to decrease between 10 and 30 min after the follicle is exposed to LH and reaches a minimum at ~1 h (13, 19). By 2 h, the permeability is beginning to return, and by 5 h, it is comparable to that before LH exposure (19). The permeability of the connexin 37 (Cx37 or GJA4) gap junctions between the cumulus cells and oocyte does not decrease during the first 5 h after LH addition (19) or injection of mice with an LH receptor agonist (42). By ~6 h after LH receptor activation, coupling between these cells decreases, due to physical separation of the cumulus cells from the oocyte by deposition of an extracellular matrix (42) and retraction of the filipodia connecting the cumulus cells and oocyte (43).

**Fig. 4.**
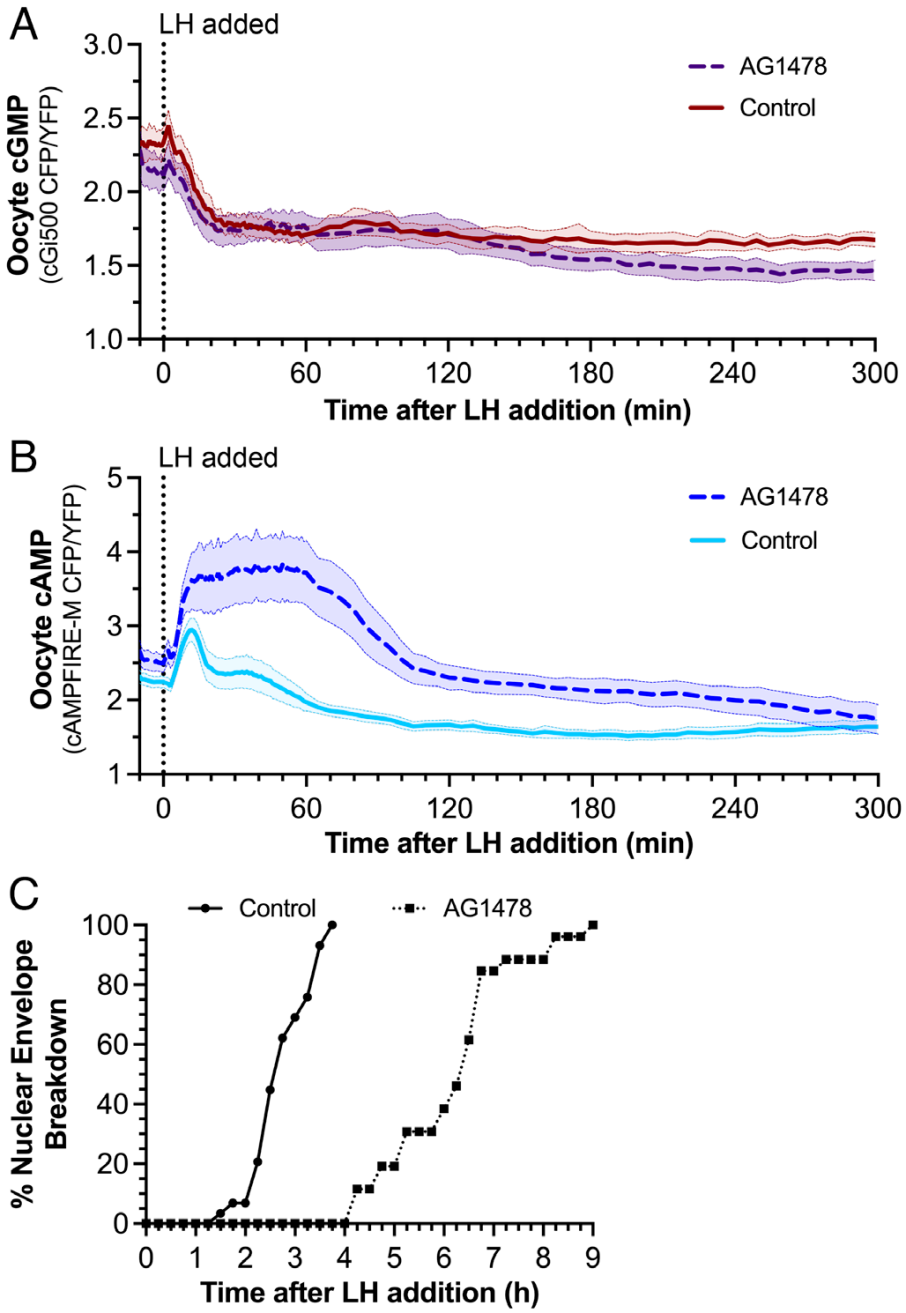
Preventing LH-induced gap junction closure does not affect cGMP dynamics but extends the oocyte cAMP elevation, delaying meiotic resumption. (*A* and *B*) LH-induced decreases in oocyte cGMP (*A*) or oocyte cAMP (*B*) were compared for control follicles or those preincubated with the EGF receptor kinase inhibitor AG1478 (0.5 µM, 1 h), which prevents LH-induced gap junction closure (44) (8 cGi500 oocytes; 17 cAMPFIRE-M oocytes; mean ± 95% confidence limit). Control data in A replotted from Fig. 3*A*; control data in B replotted from Fig. 2*C*. Note that in contrast to the results shown in Fig. 4*A*, partial effects of AG1478 on LH-induced cGMP decreases were seen in previous ELISA measurements (44, 46), possibly due to differences in experimental conditions. (*C*) Timing of LH-induced NEBD during imaging of the follicle-enclosed oocytes used for B, treated with or without AG1478. Oocytes were binned into 15-min increments for calculation of % NEBD.

LH-induced gap junction closure in the granulosa cells results from a kinase cascade that phosphorylates Cx43 (19), and LH-induced Cx43 phosphorylation and gap junction closure are reduced by the EGF receptor kinase inhibitor AG1478 (44). Under the conditions of our experiments, AG1478 had no effect on the LH-induced cGMP decrease in the oocyte (Fig. 4*A*) but did prolong the period of elevated cAMP in the oocyte, such that cAMP did not reach the same low level attained in control oocytes until ~5 h after LH addition (Fig. 4*B*). Correspondingly, AG1478 delayed NEBD by several hours (Fig. 4*C*), consistent with previous studies (44, 45). These findings show that the LH-induced closure of the Cx43 gap junctions that link the granulosa cells is essential for the normal kinetics of the oocyte cAMP decrease and meiotic resumption.

### A Working Model of Cyclic Nucleotide Dynamics in Mouse Preovulatory Follicles Before and After Exposure to LH.

**Fig. 5.**
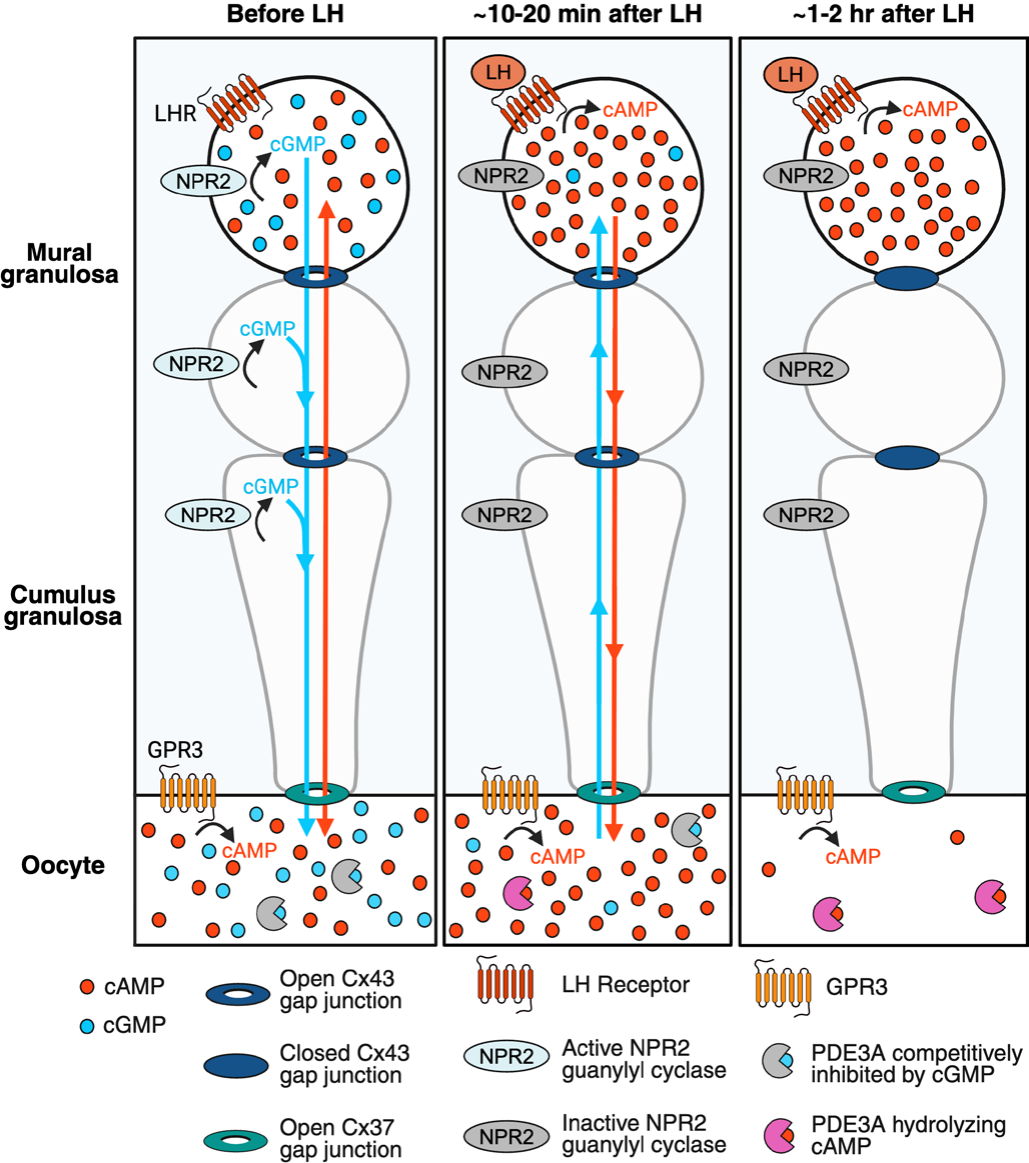
A working model of cyclic nucleotide and gap junction dynamics in mouse preovulatory follicles before and after exposure to LH. See text for explanation. Figure made in Biorender.com.

Fig. 5 shows a working model that integrates our findings with previous information (see Introduction). The distributions of cAMP (red dots) and cGMP (blue dots) in the granulosa cells and oocyte are shown before and after LH exposure. For clarity, only 3 of the ~10 layers of granulosa cells are shown, and dots representing cAMP and cGMP are shown only in the outer mural granulosa layer and in the oocyte. Both before and after LH exposure, cAMP is produced in the oocyte under the control of the constitutively active GPR3 protein. cAMP is also produced in the granulosa cells, under the control of the follicle-stimulating hormone receptor.

Before LH exposure (*Left* panel), the concentration of cAMP in the oocyte is maintained at a moderately high level, by GPR3 activity and by the diffusion of cGMP into the oocyte from the granulosa cells, which competitively inhibits the breakdown of cAMP by the PDE3A phosphodiesterase in the oocyte. cGMP is generated in the granulosa cells by the NPR2 guanylyl cyclase.

In response to LH binding to its receptors in the outer mural granulosa layer, cAMP in those cells is rapidly elevated, and cAMP diffuses to all of the granulosa cells and to the oocyte through the gap junctions (center panel). The cAMP elevation in the granulosa cells inactivates NPR2 (16, 17), and in the absence of cGMP synthesis, cGMP phosphodiesterases in the granulosa cells reduce the cGMP concentration in these cells to a low level. As a consequence, cGMP diffuses out of the oocyte into the large granulosa compartment volume, and cGMP decreases in the oocyte. When cGMP in the oocyte decreases, inhibition of PDE3A is relieved, allowing hydrolysis of cAMP.

Subsequently, the permeability of the Cx43 gap junctions between the granulosa cells decreases (*Right* panel), as a consequence of Cx43 phosphorylation. The decrease in Cx43 gap junction permeability reduces cAMP diffusion into the oocyte from the granulosa cells, and this change, together with the activation of PDE3A, causes cAMP to decrease in the oocyte. The fall in cAMP in the oocyte reinitiates meiotic progression.

### Unresolved Questions.

cAMP dynamics in the cumulus cells (Fig. 2*C* and *SI Appendix*, Fig. S3 and Movie S1) are complex. LH signaling causes cAMP to increase, then decrease transiently, then increase to the same high level as in the mural granulosa cells. Because the cumulus cells are directly connected to the oocyte by gap junctions, the transient decrease in cumulus cAMP could result from the LH-induced stimulation of cAMP phosphodiesterase activity in the oocyte. Why cAMP in the cumulus cells subsequently increases to the same level as in the mural cells is unknown. The gradual reopening of connexin 43 gap junctions between the cumulus cells and mural granulosa cells that occurs between 1 and 5 h after LH stimulation (19) could be a factor, since this would allow cAMP to diffuse into the cumulus cells from the mural granulosa cells. The cause of the oscillations that sometimes occur in the cumulus is unknown.

Another thought-provoking finding from this study is that LH signaling transiently elevates cAMP in the oocyte, before causing cAMP to fall to a level below the pre-LH baseline. Previous radioimmunassays of rabbit oocytes that were isolated into medium containing a cAMP phosphodiesterase inhibitor also showed an increase in cAMP after LH receptor stimulation of the ovary, but these results are difficult to interpret because oocyte cAMP remained above baseline through and after the time of NEBD (47). In contrast, our measurements were performed without removing the oocyte from the follicle and without a phosphodiesterase inhibitor in the medium, thus providing definitive evidence for a transient cAMP increase in the oocyte in response to LH stimulation of the granulosa cells. What then might be the physiological significance of this cAMP increase?

Studies of HEK cells have shown that cAMP elevation phosphorylates PDE3A on multiple sites, modifying the protein such that its catalytic activity is increased (48). Likewise, the transient elevation of cAMP in the oocyte might serve to further increase PDE3A activity, complementing the increase in PDE3A activity that results from the decrease in cGMP. The transient elevation of cAMP could also increase phosphorylation of other proteins in the oocyte that are important for maturation of the oocyte cytoplasm. In particular, the LH signaling pathway results in the synthesis (49) and phosphorylation (50) of IP_3_ receptors, accounting for the increase in calcium release in response to IP_3_ that occurs during oocyte maturation, as the oocyte prepares to release calcium for egg activation at fertilization (50, 51). The transient LH-induced elevation of oocyte cAMP might trigger these and other regulatory events.

## Materials and Methods

### Mice.

Mice were kept in a room with a 12-h dark/light cycle with standard diet and water ad libitum. All mouse experiments were performed according to NIH guidelines and approved by the UConn Health Center Institutional Animal Care and Use Committee.

A mouse line conditionally expressing cAMPFIRE-M was generated using CRISPR/CAS9-mediated gene editing to insert a cAMPFIRE-M expression cassette into intron 1 of *Rosa26* (*SI Appendix*, Fig. S5; see *SI Appendix*, *Supplementary Methods*). These mice were then bred with mice expressing Cre recombinase in the oocyte [*Hprt^cre^*; (52)] (obtained from the Jackson Laboratory, stock #004302, bred to C57BL/6J in our lab), resulting in mice in which cAMPFIRE-M was expressed globally (*SI Appendix*, Fig. S5). The globally expressing cAMPFIRE-M mice were maintained as homozygous breeding pairs, and homozygotes were used for all experiments. The homozygous mice were viable and fertile and showed no obvious signs of morphological or behavioral abnormalities.

*SI Appendix* Table S2 lists genotyping primers for the cAMPFIRE-M mice. Mice globally expressing cAMPFIRE-M are visibly fluorescent, such that their genotype can be determined by observation with fluorescence-detecting goggles (BLS Ltd, #FHS/T01). The conditionally expressing and globally expressing cAMPFIRE-M mice are available from Dr. Siu-Pok Yee upon request (syee@uchc.edu), and will be deposited in the Mutant Mouse Resource and Research Centers (MMRRC) repository at The Jackson Laboratory (MGI:8253176 for the conditionally expressing line; MGI:8253177 for the globally expressing line).

Mice expressing cGi500 globally were provided by Robert Feil (25); heterozygotes were used for experiments. Mice with genetically modified *Npr2* and *Pde5a* (*Npr2*-7E; *Pde5a*-S92A) were generated in our laboratory (18) and are available from Dr. Yee upon request. The background strain of all mice was C57BL/6J. Wildtype mice, obtained from The Jackson Laboratory (JAX stock #000664), were maintained as a colony in our laboratory.

### Isolation and Culture of Ovarian Follicles.

Follicles with diameters of ~320 to 360 µm were manually dissected from ovaries of 23 to 26-day-old mice. The follicles were cultured on optically clear organotypic membranes (Millicell cell culture inserts, MilliporeSigma #PICMORG50) in MEMα medium with supplements including 1 nM ovine follicle-stimulating hormone as previously described (22). The follicles were used for imaging ~24 h after dissection. An ~24-h culture period in the presence of follicle-stimulating hormone causes the expression of LH-receptors (2).

### Microinjection of Follicle-Enclosed Oocytes With mRNA Encoding cAMPFIRE-M or cGi500.

Although global expression of cAMPFIRE-M by insertion of its sequence into the *Rosa26* locus of the mouse genome resulted in sufficient protein expression for fluorescence-based measurements in the granulosa cells, the concentration of the cAMPFIRE-M protein in oocytes from these mice was lower than in the granulosa cells (*SI Appendix*, Fig. S6). This could be due to the low level of transcription in fully grown mouse oocytes (53) and/or to dilution of the transcript in the large volume of the oocyte cytoplasm. To attain sufficient expression of cAMPFIRE-M in the oocyte, additional mRNA encoding the protein was microinjected into follicle-enclosed oocytes (54), ~18 h before imaging. Likewise, for measurement of cGMP, oocytes were microinjected with mRNA encoding the cGi500 sensor. mRNA’s were transcribed in vitro (Ambion mMessage machine T7 transcription kit, Thermo Fisher #AM1344), precipitated with LiCl, then polyadenylated [Ambion poly(A) tailing kit #AM1350], and reprecipitated with LiCl. Then, 3 pg of cAMPFIRE-M mRNA, or 5 to 20 pg of cGi500 mRNA, was injected into each follicle-enclosed oocyte.

Relative to the fluorescence intensity in the mural granulosa cells, the fluorescence intensity of cAMPFIRE-M in follicle-enclosed oocytes before LH addition varied over a 5-fold range. However, the basal CFP/YFP emission ratio in the oocyte was independent of fluorescence intensity (*SI Appendix*, Fig. S7), indicating that the sensor did not change the cAMP concentration in the oocyte.

### Sources of Reagents.

Highly purified ovine FSH (AFP7558C) and ovine LH (ovine LH-26) were obtained from A.F. Parlow (National Hormone and Peptide Program, Torrance CA). The EGF receptor kinase inhibitor AG1487 was obtained from MilliporeSigma (#658552) and dissolved in DMSO. The final concentration of DMSO was 0.2%. C-type natriuretic peptide (CNP-22) was obtained from Phoenix Pharmaceuticals, Inc. (#012-03). The catalytic domain (amino acids 665-1141) of human PDE3A, purified from bacteria (6) and used for *SI Appendix*, Fig. S1, was a kind gift from Hengming Ke (University of North Carolina). Cyclic AMP used for *SI Appendix*, Fig. S1 was obtained from Enzo (#ALX-480-011).

### Imaging Methods.

For time-lapse imaging of fluorescence from cAMPFIRE-M or cGi500, up to 8 follicles were placed on 12 mm diameter Millicell cell culture inserts (Millipore-Sigma #PICM01250) from which the plastic feet were cut off. Then, 120-µm thick adhesive strips cut from Grace Bio-Labs Secure Seal imaging spacers (Millipore-Sigma #GBL 654002) were stacked to obtain 240-µm thick spacers, which were used to attach the Millicell to a 35-mm dish with a glass coverslip bottom (MatTek #P35GINV-0-20-C), creating a channel under the Millicell. To direct the flow of solution under the Millicell, silicone elastomer dams that had been made using the Millicell as a mold (Kwik-Gard Silgard 184, Electron Microscopy Sciences #24236-01) were placed around the Millicell, forming a well on either side of the channel between them (Fig. 1*D*). Then, 800 µL of medium was used to fill the wells and channel. A physiological environment was maintained by placing the dish containing the Millicell with follicles in a stage top incubation system (ibidi #10720). Humidified gas containing 5% CO_2_, 95% air at 37 °C was flowed through the incubator. Additional humidity was attained by placing wet Kimwipes in the incubator. The objective lens was kept at 37 °C using a temperature-controlled objective collar (Okolab #3342) and controller (Okolab #H401-T-PENNY).

Under these conditions, the ~400 µm diameter spherical follicles flattened to discs ~200 µm in height. The microscope was focused on the oocyte equator, ~100 µm deep within the tissue, allowing observation of the prophase-arrested nucleus (Fig. 1*B*) and its breakdown at ~2 to 4 h after LH application. NEBD was detected by the appearance of fluorescence in the space that was previously surrounded by the nuclear envelope, which excludes fluorescent proteins that are larger than ~25 kDa (Fig. 1*B*) (55). The time of NEBD was also detected by the disappearance of the nucleolus that occurs at about the same time.

Imaging was performed on a Zeiss 980 confocal microscope using a 20×/0.5 NA Plan-Neofluar objective lens to provide sufficient working distance. The cyan-emitting protein in cAMPFIRE-M or cGi500 was excited with a 445 nm diode laser (Zeiss #2183-206; 7.5 mWatt). Laser power was applied at ≤20% power (≤1.5 mWatt) to minimize light toxicity. Light from the cyan-emitting protein was collected from 450 to 514 nm, and light from the yellow-emitting protein was collected from 535 to 662 nm. Images were collected at 16-bit depth, 512 × 512 resolution, and a pixel dwell time of 4.1 µs (1.26 s total scan time). Scanning transmitted light images were collected simultaneously with differential interference contrast (DIC) optics. Images were collected once per minute for the first hour after LH addition, and once every 5 min thereafter. A computer-controlled stage allowed time-lapse imaging of up to 8 follicles in parallel.

After a brief (~10 to 30 min) equilibration period in the stage top incubator, all experiments started with at least 10 min of baseline recording. After this, LH was added by the following procedure during the ~40 to 45 s between scans. First, the microscope arm was raised to allow removal of the stage top incubator lid, and a vacuum pump was used to aspirate as much medium as possible from the imaging dish wells without touching the pipet tip aspirator to any part of the dish. Then, 800 µL of medium containing 10 nM LH was added to one well and allowed to flow through the channel under the Millicell, filling the other well. If necessary, a small volume of medium was aspirated to maintain the meniscus as close as possible to the level before perfusion. The incubator lid was then replaced gently, and the microscope arm returned to the imaging position.

### Data Analysis.

Raw time series were imported into FIJI-ImageJ (56) for initial analysis. Using the DIC image, regions of interest (ROIs) were drawn (Fig. 1*C*) and a custom macro was used to obtain the mean fluorescence values of the two channels for each ROI throughout the time series. ROIs were moved or adjusted to maintain the same sample area if the follicle shifted following perfusion or changed shape in response to LH. The fluorescence signal from the sensors was ~3 to 10 times greater than background autofluorescence, as determined for each region by averaging the signal from five wild-type uninjected follicles. After background subtraction, the sensor signal was corrected for spectral overlap, by subtracting 23% of the intensity in the CFP channel from the intensity in the YFP channel.

Movie S1 was generated in FIJI-ImageJ by first registering the time series using the plugin HyperStackReg (57). The channels were then separated and subjected to background and spectral overlap subtraction, formatted to 32 bit, 2x2 binned, and then combined into a single CFP/YFP time series using the “Image Calculator” function in FIJI-ImageJ. The area outside the follicle was masked for clarity. The scale bar and CFP/YFP calibration bar were added in FIJI-ImageJ; other annotations and editing were performed using www.veed.io.

Statistical analyses were performed as described in the figure legends using Prism 10 (GraphPad).

## Supplementary Material

Appendix 01 (PDF)

Movie S1. Time lapse imaging of LH-induced cAMP changes in a preovulatory follicle from a mouse globally expressing cAMPFIRE-M, with additional cAMPFIRE-M mRNA injected into the oocyte, during the 3-hour period after LH perfusion. Images were collected at 1-minute intervals for 10 minutes before LH addition, and for 60 minutes afterwards; images were then collected at 5-minute intervals between 60 and 180 minutes. Frames from Movie S1 are shown in Fig. 2*D*.

## Data Availability

All study data are included in the article and/or supporting information.
