# Supplementary material for: Intercellular diffusion of cyclic nucleotides followed by gap junction closure restarts meiosis in mouse preovulatory follicles
Source: Proc Natl Acad Sci U S A. 2025 Dec 2;122(49):e2524136122. doi: 10.1073/pnas.2524136122 (PMC12704752; doi:10.1073/pnas.2524136122)
Supplement: Supplementary file 1 — Appendix 01 (PDF) [file pnas.2524136122.sapp.pdf]

PNAS

Supporting Information for
Intercellular diffusion of cyclic nucleotides followed by gap junction closure restarts meiosis in mouse preovulatory follicles.

Iris F. Nakashima, Haining Zhong, Viacheslav O. Nikolaev, Corie M. Owen, Siu-Pok Yee, Laurinda A. Jaffe, and Jeremy R. Egbert

This PDF file includes:

Supporting text
Figures S1 to S7
Tables S1 to S2
Legend for Movie 1
SI References

Other supporting materials for this manuscript include the following:

Movie S1

Supporting Text

Generation of cAMPFIRE-M Expressing Mouse Lines. Mice were generated by the Center for Mouse Genome Modification (CMGM) at UConn Health, using CRISPR/Cas9 mediated gene editing. The design of the donor vector was similar to that of Ai9 (Addgene plasmid #22799) as described by (1). The Rosa26 conditional expression vector was prepared by PCR using Ai9 as template and conventional molecular cloning methods to introduce a unique AsiSI restriction site between the LoxP-STOP-LoxP and WPRE sequence as described previously (2, 3). The coding sequence of cAMPFIRE in the pCDNA3_cAMPFIRE-M plasmid (Addgene plasmid #182280) is flanked by unique restriction sites, HindIII and XbaI. These two sites were converted into AsiSI by restriction digestion followed by insertion of Hind-to-AsiSI oligo (5'-AGC TAT AGC GAT CGC TAT) and Xba-to-AsiSI oligo (5'- CTA GTA TGC GAT CGC ATA), respectively. The cAMPFIRE-M sequence was then released by AsiSI digestion followed by insertion into the unique AsiSI site in the Rosa26 expression vector. The proper orientation of the cAMPFIRE-M sequence in the vector was confirmed by restriction digestions and sequencing.

Guide RNA sequence, 5′-GAA GAU GGG CGG GAG UCU UC, specific to Rosa26 intron 1 was identified using Chopchop (https://chopchop.cbu.uib.no). Mice were generated by pronuclear microinjection of one-cell embryos isolated from C57BL/6J mice (JAX stock #000664) with donor vector (20 ng/μl) and CRISPR/Cas9 RNP (100 ng/μl Cas9 protein, 50 ng/μl crRNA, 100 ng/μl tracrRNA). Injected embryos were then transferred into CD1 pseudo-pregnant foster females for subsequent development. Potential founders were initially screened for the presence of the expression cassette and then confirmed by nested long-range PCR using primers specific to the expression cassette and outside the homology arms of the donor vector. For the 5'-homology arm, we used the primer pairs R26 I1F1 (5′- GCT AGG TAG GGG ATC GGG ACT C) and AiCAGR1A (5′- GGC GTT ACT ATG GGA ACA TAC GTC), followed by R26 I1F2 (5′- CTT GGT GCG TTT GCG GGG ATG) and AiCAGR1A, with an anticipated PCR product of 1.2 kb. For the 3'-homology arm, we first used the primer pairs RGpA5F1 (5′- CCT CCT CTC CTG ACT ACT CCC AG) and RosaE2R1 (5′- CCT CCT CTC CTG ACT ACT CCC AG), followed by RBGpAf (5′- CTC CCA GTC ATA GCT GTC CCT C) and nR26E2R2 (5′- GCC TTA AAC AAG CAC TGT CCT GTC C), to detect an expected PCR product of 4.7 kb. Confirmed positive founders were bred with wildtype animals and positive F1 pups were further confirmed using both cassette-specific primer pairs followed by nested long-range PCR.

Positive F1 pups were bred with *Hprt*-Cre mice (JAX stock #004302), which had been backcrossed with C57BL/6J for over 30 generations, to establish a mouse line with global expression of cAMPFIRE-M in C57BL/6J background. Primer sequences and specificities for subsequent routine genotyping are shown in **Table S2** and the breeding strategy is shown in **Figure S5**.

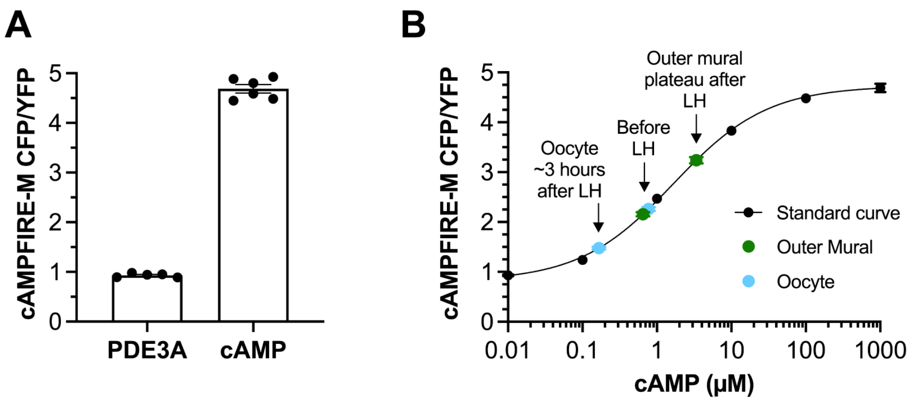

C

$$Y = 0.81 + \frac{X^{0.69}(4.73 - 0.81)}{X^{0.69} + 1.67^{0.69}}$$

Where Y = cAMPFIRE-M CFP/YFP
and X = [cAMP]

Figure S1. In vivo calibration of the cAMPFIRE-M sensor to estimate cAMP concentrations in oocytes and mural granulosa cells within intact follicles. (A) cAMPFIRE-M CFP/YFP ratios were measured in follicle-enclosed oocytes after microinjection of 200 μg/ml of the catalytic domain of a high-affinity cAMP phosphodiesterase (PDE3A, K_m ~90 nM) (4) to lower cAMP to a minimum, or microinjection of 1 mM cAMP to raise cAMP to a maximum (mean ± SEM). (B) Values determined in (A) were used to define the minimum and maximum points of the standard curve. Data from a standard curve for cAMPFIRE-M in vitro (5) were then scaled to connect the minimum and maximum points. (C) An equation describing the curve in (B) was generated using sigmoidal 4-parameter logistic regression (Prism 10). The equation in (C) was then used to estimate average cAMP concentrations from CFP/YFP measurements from oocytes and mural granulosa cells before and after LH application (data from **Figure 2C**). The results of these calculations are plotted on the graph in (B). Blue points indicate oocyte values and green points indicate outer mural granulosa values (mean ± SEM).

Methods for Figure S1

Preovulatory follicle-enclosed oocytes from wild-type mice were injected with cAMPFIRE-M mRNA and cultured overnight to allow protein expression (see Materials and Methods in main text). ~18 hours later, follicle-enclosed oocytes were injected with either 10 pl of 4 mg/ml PDE3A catalytic domain protein (200 μg/ml concentration in oocyte) (n = 5 follicles), or 10 pl of 20 mM cAMP (1 mM concentration in oocyte) (n = 6 follicles). After injection, the follicles were removed from the injection chamber and plated on the Millicell of an imaging dish, for measurement of CFP/YFP ratios. For PDE3A-injected oocytes, cAMPFIRE-M ratios were measured ~15 min after injection; ratios ~40 min after injection were similar, indicating that a minimum cAMP concentration was attained by 15 minutes. For cAMP-injected oocytes, similar ratios were obtained when imaged as quickly as possible following injection (4-6 min) or ~15 min after injection. However, ratios had declined by 40 min after cAMP injection. For the graph shown in B, the standard curve was generated assuming that the minimum [cAMP]

attained after PDE3A injection was 10 nM. The results of the calibration were similar if we assumed that the [cAMP] attained after PDE3A injection was 1 nM.

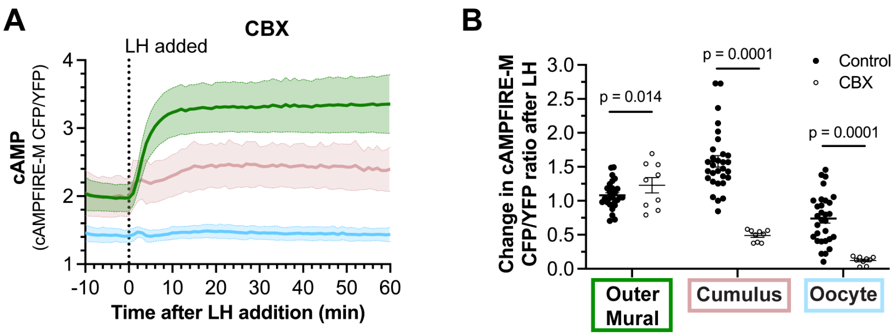

Figure S2. Gap junction-dependence of basal cAMP in the oocyte, and of LH-induced propagation of cAMP from the mural granulosa cells to the cumulus cells and oocyte. Follicles were incubated for 2 hours with the gap junction-inhibitor carbenoxolone (CBX, 200 μM) before measuring LH-induced cAMP changes; n = 13 follicles. (A) shows the CFP/YFP emission ratio in each region before and after LH addition (mean ± 95% confidence limit). (B) shows the maximum LH-induced increase in CFP/YFP ratio in each region, with or without CBX pre-incubation (control data from Figure 2; mean ± SEM); p-values from 2-way ANOVA followed by the Sidak correction for multiple comparisons.

These results show that basal cAMP levels in the mural and cumulus cells were not changed by inhibition of gap junction communication, but basal cAMP in the oocyte decreased (compare Figure S1A with control data in Figure 2A). This decrease in basal cAMP in the oocyte presumably occurs because in the absence of cGMP diffusion into the oocyte from the granulosa cells, the PDE3A phosphodiesterase is activated in the oocyte (6). The presence of CBX also prevented the LH-induced increase in cAMP in the oocyte (Figures 2A and S2A,B). With CBX present, the LH-induced cAMP increase in the cumulus cells was attenuated, while in the mural granulosa cells the cAMP increase was enhanced (Figure S2B), consistent with the restriction of cAMP diffusing from the mural cells to the cumulus. These measurements indicate that gap junctions mediate at least most of the LH-induced inward propagation of the cAMP increase. The residual cAMP increase in the cumulus cells in the presence of CBX could be due to incomplete inhibition of gap junction permeability by CBX, or to alternative mechanisms.

A

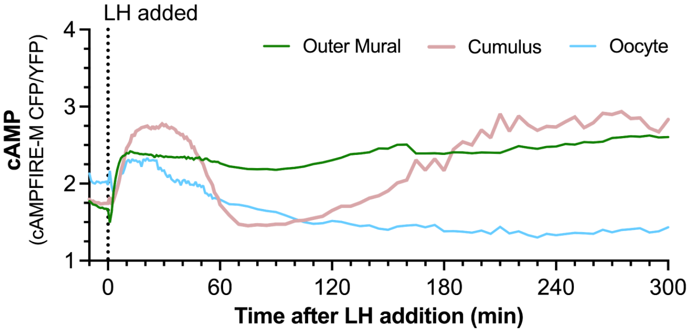

B

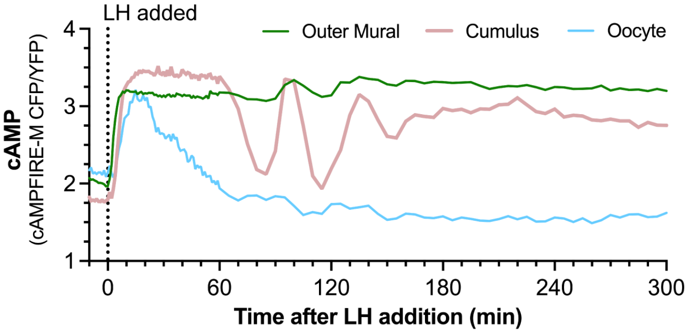

Figure S3. Variable oscillations in the CFP/YFP emission ratio in the cumulus cells between 1 and 3 hours after LH addition. (A) An example in which the LH-induced cAMP increase in the cumulus region was followed by a transient decrease and then a gradual increase to a plateau at a high cAMP level similar to that in the mural granulosa region. (B) An example in which the LH-induced cAMP increase in the cumulus region was followed by oscillations before the plateau at a high level of cAMP was established. This example is from the follicle for which oocyte and mural granulosa cell traces are shown in Figure 2D.

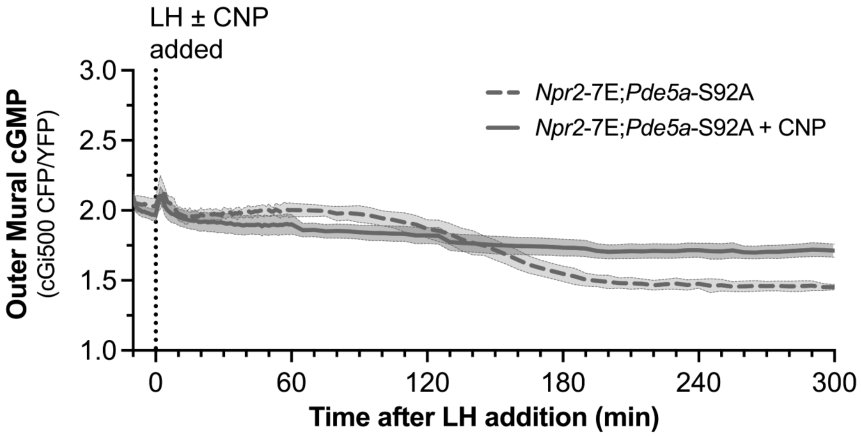

Figure S4. Exogenous CNP prevents most of the remaining LH-induced cGMP decrease in the outer mural granulosa cells of follicles from *Npr2*-7E;*Pde5a*-S92A mice. These data support the hypothesis that the delayed cGMP decrease 2-3 hours after LH in *Npr2*-7E;*Pde5a*-S92A follicles is due to an LH-induced decrease in follicle CNP content. Following a 10-min baseline recording, *Npr2*-7E;*Pde5a*-S92A follicles were either treated with 10 nM LH only (dashed line; n = 8) or with LH + 1 *μ*M CNP (solid line; n = 11) as detailed in the main text Materials and Methods (mean ± 95% confidence limit).

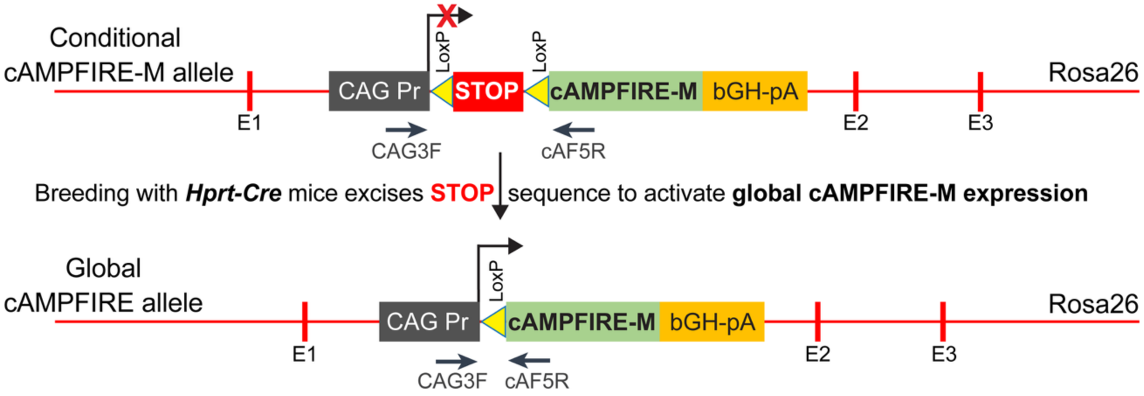

Figure S5. cAMPFIRE-M mouse design. The conditional cAMPFIRE-M allele was targeted to the mouse *Rosa26* locus and global cAMPFIRE-M expression was activated by breeding with *Hprt-Cre* mice. CAG Pr = synthetic promoter composed of cytomegalovirus early enhancer element and chicken beta-actin promoter. bGH-pA = bovine growth hormone polyadenylation signal. CAG3F: Forward genotyping primer near the 3' end of the CAG promoter sequence. cAF5R: Reverse genotyping primer near the 5' end of the cAMPFIRE-M sequence. E1-E3: Exons of the *Rosa26* locus.

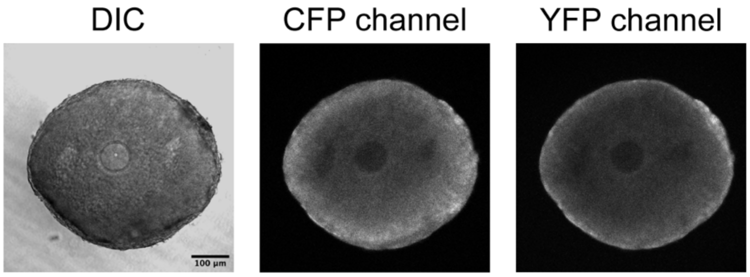

Figure S6. Distribution of cAMPFIRE-M protein in a follicle from a mouse expressing cAMPFIRE-M globally, without injection of cAMPFIRE-M mRNA into the oocyte. The low level of fluorescence in the oocyte indicates the low level of cAMPFIRE-M protein in the oocyte relative to that in the granulosa cells.

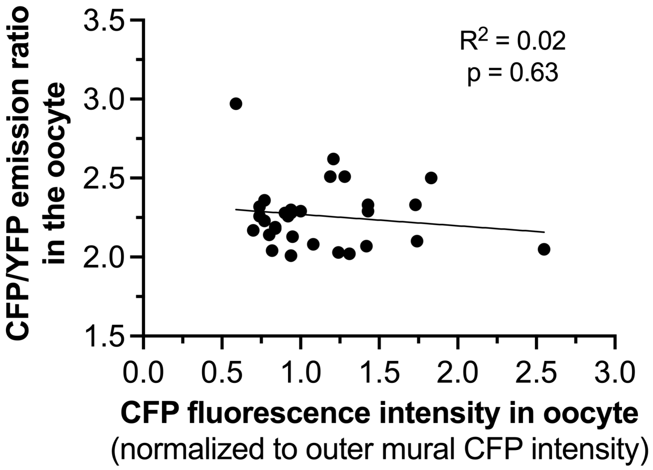

Figure S7. The cAMPFIRE-M sensor did not change the basal cAMP concentration in the oocyte. Injection of mRNA encoding cAMPFIRE-M into follicle-enclosed oocytes resulted in protein expression levels that varied over a 5-fold range among the 30 follicles used to obtain the data in Figure 2A. The graph in Figure S7 shows no correlation between the amount of cAMPFIRE-M protein in the oocyte and the basal cAMP concentration in the oocyte, as measured before LH addition. The x-axis shows the amount of cAMPFIRE-M protein in the oocyte as indicated by the CFP fluorescence intensity in the oocyte normalized to the CFP fluorescence intensity in the mural granulosa cells. The y-axis shows the concentration of cAMP in the oocyte before LH addition as indicated by the cAMPFIRE-M CFP/YFP emission ratio. Data were analyzed by simple linear regression; coefficient of determination (R^2) and p-value for the test that the slope is significantly different from zero are reported.

Table S1. LH-induced increases in mural granulosa cell CFP/YFP emission ratios in ovarian follicles from mice expressing cAMPFIRE-M vs Epac-camps sensors.

Mouse line	EC_{50} of sensor for cAMP	% increase in CFP/YFP ratio after applying LH[c]
cAMPFIRE-M (*Rosa26* knock-in)[a]	1.4 μM[5]	50 ± 9 (30 follicles)
Epac1-camps (random transgenesis)[7]	2.4 μM[9]	10 ± 2 (18 follicles)
Epac1-camps (*Rosa26* knock-in)[8]	2.4 μM[9]	12 ± 2 (7 follicles)
Epac2-camps300 (*Rosa26* knock-in)[b]	0.3 μM[6]	9 ± 3 (4 follicles)

[a]This study

[b]Mice were generated as described in (8).

[c]Values indicate the % increase (mean ± SD) from the baseline before LH addition to the peak level attained after addition of LH. The baseline was defined as the average value for the 10 minutes prior to LH administration, and the peak was defined as the average for the highest 3 sequential scans in the first 60 minutes after LH. Measurements using cAMPFIRE-M were made as described in the Materials and Methods. Measurements using other sensors were made as described by (10). Excitation and emission wavelengths, and methods for correcting for autofluorescence and spectral bleed-through of CFP into the YFP channel (6) were similar for all measurements.

Table S2. Primers and protocol for genotyping of cAMPFIRE-M mice.

**Target Region**	**Primer Name**	**Primer Sequence**	**Size of amplicon**	**Anneal Temp. °C**
5’ insert	LxFse	5’- CCTCGACCTGCAGCCCAAGC	142 bp	64
	cAF5R	5'- CGC CGT CCA GCT CGA CCA GG		
3’ insert	cAF3F	5'- ATC GAG CCG AAT TCC CTC GAG G	180 bp	58
	R26-WHVER	5’- GCGTATCCACATAGCGTAAAAGGAGC		
After Cre excision	CAG3F	5’- GGGTTCGGCTTCTGGCGTGTG	337 bp after excision (1.2 kb before excision)	63
	cAF5R	5'- CGC CGT CCA GCT CGA CCA GG		
WT allele	R26-TDF	5’- CTCTGCTGCCTCCTGGCTTCTGAG	325 bp	61
	R26-TDR	5’- CTCCGAGGCGGATCACAAGC		

Genomic DNA for genotyping was prepared from ear notches using the hotshot method (11). Genotyping was performed using one μl of genomic DNA in a 15 μl reaction mix (Jumpstart REDTaq ReadyMix, Sigma) according to the manufacturer's instructions.

Cycling parameters

Temperature	Time	Cycles
94°C	3min	1
94°C	30 sec	35
Anneal	30 sec	
72°C	60 sec	
72°C	5 min	1
12°C	Indefinitely	

Legend for Movie S1. Time lapse imaging of LH-induced cAMP changes in a preovulatory follicle from a mouse globally expressing cAMPFIRE-M, with additional cAMPFIRE-M mRNA injected into the oocyte, during the 3-hour period after LH perfusion. Images were collected at 1-minute intervals for 10 minutes before LH addition, and for 60 minutes afterwards; images were then collected at 5-minute intervals between 60 and 180 minutes. Frames from Movie S1 are shown in Fig. 2D.

SI References

1. L. Madisen, *et al.*, A robust and high-throughput Cre reporting and characterization system for the whole mouse brain. *Nat Neurosci* **13**, 133–140 (2010).

2. L. M. Mehlmann, T. F. Uliasz, S.-P. Yee, D. Kaback, K. M. Lowther, Generation and Characterization of a TRIM21 Overexpressing Mouse Line. *Genesis* **62**, e23616 (2024).

3. H. C. Hrdlicka, *et al.*, Inhibition of miR-29-3p isoforms via tough decoy suppresses osteoblast function in homeostasis but promotes intermittent parathyroid hormone-induced bone anabolism. *Bone* **143**, 115779 (2021).

4. R. Hambleton *et al.*, Isoforms of cyclic nucleotide phosphodiesterase PDE3 and their contribution to cAMP hydrolytic activity in subcellular fractions of human myocardium. *J Biol Chem* **280**, 39168-39174 (2005).

5. C. I. Massengill, *et al.*, Sensitive genetically encoded sensors for population and subcellular imaging of cAMP in vivo. *Nat Methods* **19**, 1461–1471 (2022).

6. R. P. Norris, *et al.*, Cyclic GMP from the surrounding somatic cells regulates cyclic AMP and meiosis in the mouse oocyte. *Development* **136**, 1869–1878 (2009).

7. D. Calebiro, *et al.*, Persistent cAMP-signals triggered by internalized G-protein-coupled receptors. *PLoS Biol* **7**, e1000172 (2009).

8. K. Špiranec, *et al.*, Endothelial C-Type Natriuretic Peptide Acts on Pericytes to Regulate Microcirculatory Flow and Blood Pressure. *Circulation* **138**, 494–508 (2018).

9. V. O. Nikolaev, M. Bünemann, L. Hein, A. Hannawacker, M. J. Lohse, Novel single chain cAMP sensors for receptor-induced signal propagation. *J Biol Chem* **279**, 37215–37218 (2004).

10. L. C. Shuhaibar, *et al.*, Intercellular signaling via cyclic GMP diffusion through gap junctions restarts meiosis in mouse ovarian follicles. *Proc Natl Acad Sci U S A* **112**, 5527–5532 (2015).

11. G. E. Truett, *et al.*, Preparation of PCR-quality mouse genomic DNA with hot sodium hydroxide and tris (HotSHOT). *Biotechniques* **29**, 52, 54 (2000).
